# circRNA18_46222157_46248185 inhibits melanogenesis by targeting miR-211/*EP300* pathway in goat melanocytes

**DOI:** 10.5713/ab.24.0316

**Published:** 2024-08-26

**Authors:** Kai Yuan Ji, Xue Qing Zhang, Yi Wei Zhao, Chun E Liang, Xin Yuan, Yun Hai Zhang

**Affiliations:** 1 Anhui province Key Laboratory of Genetic Resources Protection and Biological Breeding for Livestock and Poultry, College of Animal Science and Technology, Anhui Agricultural University, Hefei 230036, China; 2 Linquan Comprehensive Experimental Station of Anhui Agricultural University, Anhui Agricultural University, Linquan 236400, China

**Keywords:** *Capra hircus*, circRNA, Melanocyte, Melanogenesis, miRNA

## Abstract

**Objective:**

This study investigated the effects of circRNA18_46222157_46248185 (named circRNA18) on goat melanogenesis, which differs significantly in goat skins isolated from white and brown coat-colored skins.

**Methods:**

Expression patterns of circRNA18 in goat skin and melanocytes were determined by quantitative real-time polymerase chain reaction (qRT-PCR) and *in situ* hybridization. The circRNA18 interference vector was designed and synthesized to transfect melanocytes and detect the effect of circRNA18 interference on melanin production. Bioinformatics software was used to predict the targeted adsorption miRNAs of circRNA18, verified by luciferase assay. A miRNA expression vector was constructed and transfected into melanocytes to detect the effect of miRNA on melanin production, and the targeted regulatory genes were detected by luciferase assay. Target gene interference vector was constructed to detect the influence of target gene interference on melanin production.

**Results:**

qRT-PCR results unveiled distinct expression patterns of circRNA18 in diverse tissues of male and female goats, while *in situ* hybridization assays showed that circRNA18 is expressed in the cytoplasm of melanocytes. Functional analysis demonstrated that the downregulation of circRNA18 in melanocytes leads to a significant increase (p<0.01) in melanin production. Bioinformatics analysis identified a potential miR-211 binding site on circRNA18, and luciferase assay confirmed their interaction. Overexpression of miR-211 in melanocytes significantly augmented (p<0.01) melanin production. There were two potential miR-211 binding sites on adenoviral E1A-binding protein (*EP300*), and the overexpression of miR-211 in melanocytes significantly decreased (p<0.001) *EP300* expression, with luciferase assay confirming their interaction. Downregulation of *EP300* expression in melanocytes through siRNA-*EP300* transfection results in a substantial increase (p<0.05) in melanin production. qRT-PCR results indicated that overexpression of mimics-circRNA18 in melanocytes markedly suppressed (p<0.0001) miR-211 expression, significantly elevated (p<0.01) *EP300* expression, and significantly inhibited (p<0.001) melanin production.

**Conclusion:**

circRNA18_46222157_46248185 acted as a negative regulator of melanogenesis in goat melanocytes by targeting the miR-211/*EP300* pathway, and guiding animal hair color breeding strategies.

## INTRODUCTION

Avian species also are important for color breeding related to reproduction and ecological adaptations. Previous studies have shown that coat color in mammals is determined by tyrosine-derived pigments (eumelanin and brown melanin) and that the spatial and temporal distribution of different types of melanin particles results in different patterns and skin colors [1]. Melanocytes are located in the skin epidermis and at the base of hair follicles and contain an acidic organelle called the melanosome. Mature melanosomes produce melanin particles through tyrosinase catalysis [2]. Studies on humans and mice have shown that more than 100 loci are involved in melanogenesis [3,4]. MITF is a basic leucine-zipper transcription factor that binds to the CATGTG sequence in the promoter region of the tyrosine gene family and regulates its expression [5]. Previous studies have confirmed that MITF is widely involved in the melanocyte proliferation, differentiation, and melanogenesis [6,7]. However, the mechanism of mammalian coat color formation is complex and remains unclear.

MicroRNAs (miRNAs) are evolutionarily conserved non-coding RNA molecules that play important roles in the regulation of gene expression in eukaryotes [8,9]. Previous studies have confirmed that multiple miRNAs involved in melanogenesis in mammals [4,10]. Among these, miR-101a-3p and miR-144a-3p downregulate pigmentation by co-targeting microphthalmia-associated transcription factors in alpaca melanocytes [11], and microRNA-379 mediates pigmentation, migration, and proliferation of alpaca melanocytes by targeting the insulin-like growth factor 1 receptor [12]. However, the mechanisms underlying the activities of miRNAs in the development of mammalian pigmentation have yet to be elucidated, and the factors influencing miRNA expression and function in melanocytes remain unclear.

Circular RNA (circRNA) is a non-coding RNA molecule that has no 5′-end cap or 3′-end Poly (A) tail and forms a ring structure with covalent bonds [13]. Compared with traditional linear RNAs, it is not easily degraded by the exonuclease RNase R and is more stable than linear RNAs [13]. circRNAs modulate the expression of downstream genes by targeting miRNAs adsorption [12,14,15]. Previous studies have shown that circRNA is involved in many biological processes and has been extensively explored [16,17]. Recent studies have implicated that circRNAs are involved in melanogenesis in mice [18], however, their specific roles are poorly understood.

While the involvement of circRNAs and miRNAs in disease pathogenesis, cellular metabolism, and various physiological processes has been well established, the mechanisms underlying the interplay of circRNA-miRNA interactions in skin pigmentation in livestock remain inadequately elucidated. Whole transcriptome sequencing analysis has unveiled that circRNA18_46222157_46248185 (named circRNA18) is more abundant in goat skin from white-coated than in those from brown-coated (PRJNA995898). The analysis predicted potential binding sites between circRNA18 and miRNAs (miR-211 and miR-7-5p) involved in pigmentation [19,20]. Building upon these findings, we postulate that circRNA18 may be involved in goat skin pigmentation as a ceRNA.

## MATERIALS AND METHODS

### Ethics approval

The experimental animals were obtained from Linquan Comprehensive Experimental Station of Anhui Agricultural University. The study was conducted according to the guidelines of the Declaration of Helsinki and approved by the Ethics Committee of Anhui Agricultural University. The Institutional of Animal Care and Use Committee of Anhui Agricultural University approved all animal surgeries (Approval no. AHAUXMSQ2023065).

### Experimental design

The goat melanocytes used in this study were established in our laboratory and maintained as previously described [21]. In the present research, we investigated the role of circRNA18_46222157_46248185 (named circRNA18) on melanogenesis at the cellular level. The research was divided into seven experiments. Each experiment had a completely randomized design, and was conducted in triplicate. Experiment 1 was designed to elucidate the expression pattern of circRNA18 in goat skins and melanocytes, including location, expression trend, etc. Experiment 2 soughted to unveil the effect of circRNA18 on melanogenesis by overexpression of siRNA-circRNA18 plasmid and negative control (NC) plasmid in melanocytes. Experiment 3 aimed to delineate the regulatory interplay between circRNA18 and specific miRNAs (miR-211 and miR-7-5p) through luciferase assay. Experiment 4 was conducted to investigate the effects of miR-211 on melanogenesis by overexpression of miR-211 plasmid in melanocytes. Experiment 5 aimed to uncover the regulatory relationship between miR-211 and E1A-binding protein p300 (*EP300*) in melanocytes through luciferase assay. Experiment 6 was devised to explore the impact of *EP300* on melanogenesis by overexpression of siRNA-*EP300* plasmid in melanocytes. Experiment 7 aimed to elucidate the molecular mechanism by which circRNA18 inhibits melanin production through the investigation of overexpressing mimics-circRNA18 plasmid.

### Skin sample collection and RNA isolation

Experimental animals were obtained from the Linquan comprehensive experimental station of Anhui Agricultural University. Skin samples were obtained from the back skin (0.8×0.8 cm) of three 3-month-old Boer goats. The goats were provided adequate drinking water and feed during the experiment to maintain good health. Total example RNAs were extracted using the TRIzol method, and the total sample RNAs were marked and stored at −80°C.

### Functional analysis

Circular RNAs have the potential to function as miRNA sponges, thereby impacting miRNA activity in the modulation of mRNA expression [14,23]. In this study, we predicted the potential target miRNAs of circNA18 using the TargetScanVert and miRDB databases. miRNAs can regulate gene expression by binding it’s 3′-untranslated regions (UTR) [22,23]. In this study, we predicted the potential target genes of miRNA (miR-211) using the TargetScanVert and miRDB databases.

### Quantitative real-time polymerase chain reaction

Total RNA was extracted from goat skin samples and melanocytes using the TRIzol reagent, and cDNA was synthesized using a high volume cDNA reverse transcription kit (TransGen, Beijing, China). The cDNA amplification was conducted utilizing the PCR system (Thermo Fisher, Waltham, MA, USA) with specific primers (Table 1). The expression levels of genes (miRNA and circRNA) were normalized to universal U6 primer (U6) and glyceraldehyde-3-phosphate dehydrogenase (*GAPDH*), respectively (n = 3, mean±standard deviation [SD]). Relative expression levels of genes were calculated using the 2^−ΔΔCt^ method.

### Plasmid construction

In order to explore the functions of circRNA18 in melanocytes, siRNA-circRNA18 vectors and the NC vectors were chemically synthesized (Beijing Genomics institution, Shenzhen, China). The precursor sequence of potential target miR-211 was obtained from the miRBase database (http://www.mirbase.org) and chemically synthesized, then inserted into the pcDNA6.2-GW/EmGFPmiR vector (Invitrogen, Carlsbad, CA, USA). A segment of cDNA fragment of circRNA18, encompassing binding sites for miR-211, was amplified and subsequently subjected to mutation via chemical synthesis. These amplified fragments were cloned into the pmirGL0 Dual-Luciferase miRNA target vector (Promega, Madison, WI, USA) to generate the pmirGL0-circRNA18-wt and pmirGL0-circRNA18-mut plasmids. Additionally, a mimics-circRNA18 plasmid, with binding sites for miR-211, and a NC plasmid were also developed.

### Cell culture and transfection

Goat melanocytes were cultured in melanin basal medium (ScienCell Research Laboratories, Carlsbad, CA, USA). HEK 293T cells were cultured in Dulbecco’s modified eagle medium (Gibco, New York, NY, USA) supplemented with 10% fetal bovine serum (Kangyuan, Shanghai, China). The cells employed in this investigation were transfected using LipofectamineTM 2000 (Invitrogen, USA).

### Tyrosinase activity determination

Counted the melanocytes, and the melanocytes were added to 90 μL of Triton X-100 (1%) and shaken for 5 min. Thereafter, 5 μL of 0.2% L-dopa was added and the cells were incubated at 37°C for 30 min. Optical density (OD) was measured at 490 nm using a microplate analyzer (Biotek, Winooski, VT, USA), and tyrosinase activity (%) was calculated according to the manufacturer’s protocol.

### Melanin content measurements

Melanin production was analyzed using a total alkali-soluble melanin (ASM) assay. Melanocytes were collected and counted, rinsed three times with phosphate-buffered saline (PBS) (1×), and lysed in 1 mL of NaOH (1 mol/L) at 37°C for 45 min. The OD was measured at 475 nm and normalized against the cell number. Melanin production (%) was calculated according to manufacturer’s protocol.

### Luciferase assay

HEK 293T cells were co-transfected with 3 μg of pmirGL0-circRNA18-wt or pmir-GL0-circRNA18-mut together with an miR-211 plasmid. Cells were lysed after 48 h using the lysis buffer supplied with the Dual-Luciferase Assay Kit (Beyotime, Shanghai, China), and firefly and Renilla luciferase activities were measured. Luciferase activity (%) was calculated based on OD measurements.

### *In situ* hybridization

The goat melanocytes were inoculated on slides, fixed in 4% paraformaldehyde for 30 min at 4°C, and treated with proteinase K (0.04 μg/mL; Roche Applied Science, Shanghai, China). The samples were washed with PBS (0.1 mol/L, pH = 7.4) and hybridized with circRNA 18_46222157_46248185 probe (5′-ACACCTACAATACCTCAACGACTATTACT GCCTCTT-3′; Bio-High, Shijiazhuang, China) at 37°C overnight. The slides hybridized with the probe were washed in saline sodium citrate solution, and incubated with mouse alkaline phosphatase (ALP)-conjugated anti-digoxigenin antibody (1:1,000; Bio-High, China) for 2.5 h at 37°C. The ALP reaction was performed using 3,3′-diaminobenzidine as the substrate.

### Statistical analysis

Data were analyzed using GraphPad Prism software (version 8.0). Data, including tyrosinase activity, melanin production, luciferase activity, and gene abundance, were determined using Student’s *t*-test. Data were arcsine-transformed prior to statistical analysis, and differences among means were assessed using the least significant difference method. A single replicate was used as the experimental unit. Unless indicated, the results were expressed as the mean±SD, and values were considered statistically significant at the p<0.05 level.

## RESULTS

### Expression pattern of circRNA18_46222157_46248185

**Figure 1:**
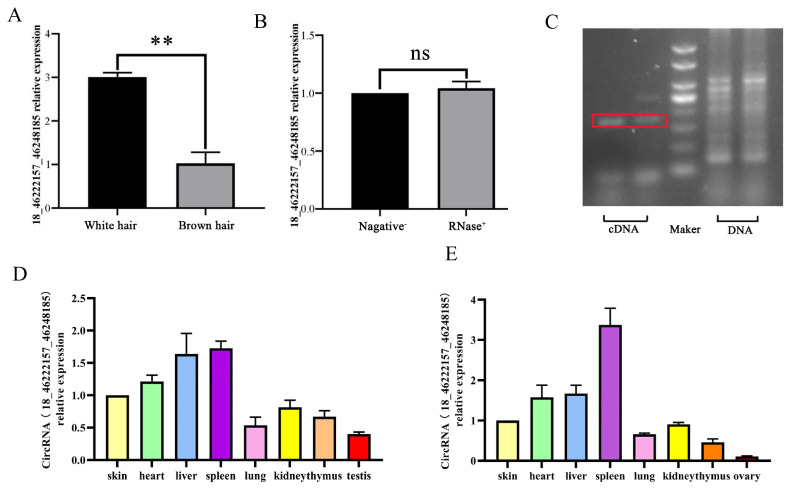
CircRNA18_46222157_46248185 (named circRNA18) expression pattern. (A) The expression trend of circRNA18 in goat skins with white and brown hair. (B) The effect of RNA enzyme on circRNA18 expression (Negative-stand for unpurified, RNase+ means it has been purified by RNase). (C) The source of circRNA18 detected using polymerase chain reaction. The expression profile of circRNA18 in various tissues of male goat (D) and female goat (E). Data represent mean±standard deviation of the relative fold-change (n = 3 per group). ** p<0.01, ^ns^ p>0.05.

Quantitative real-time polymerase chain reaction (qRT-PCR) results showed that circRNA18 expression level in skin with white hair was significantly higher (p<0.01) than that with brown hair (Figure 1A), and the expression of cicrcRNA18 was not affected by RNA enzyme treatment (Figure 1B). The PCR results showed no positive signal in the DNA of melanocytes using specific primers, but a specific positive signal was detected in the cDNA (Figure 1C). The results of the circRNA expression profile showed distinct expression patterns of circRNA18 in diverse tissues of male (Figure 1D) and female (Figure 1E) goats, and they were highly abundant in the spleen and liver.

### Localization of circRNA18_46222157_46248185

**Figure 2:**
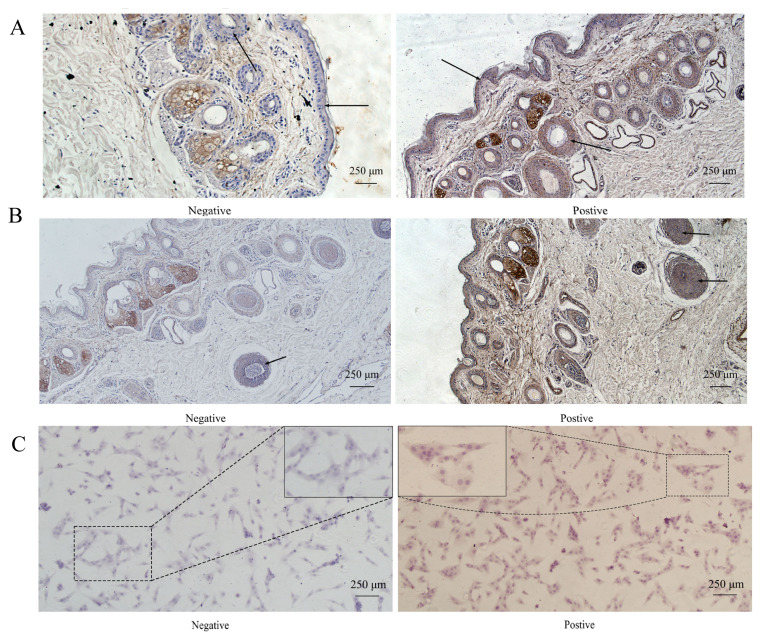
Location of circRNA18_46222157_46248185 (named circRNA18) in goat skin and melanocytes. *In situ* hybridization results revealed circRNA18 expression in the basal layer of skin (A), hair follicles (B), the cytoplasm of melanocytes (C).

The *in situ* hybridization results revealed that circRNA18 was expressed in the basal layer of the skin (Figure 2A) and hair follicles (Figure 2B), where melanocytes reside. Moreover, we observed ubiquitous expression of circRNA18 in the cytoplasm of melanocytes (Figure 2C).

### CircRNA18_46222157_46248185 inhibits melanogenesis

**Figure 3:**
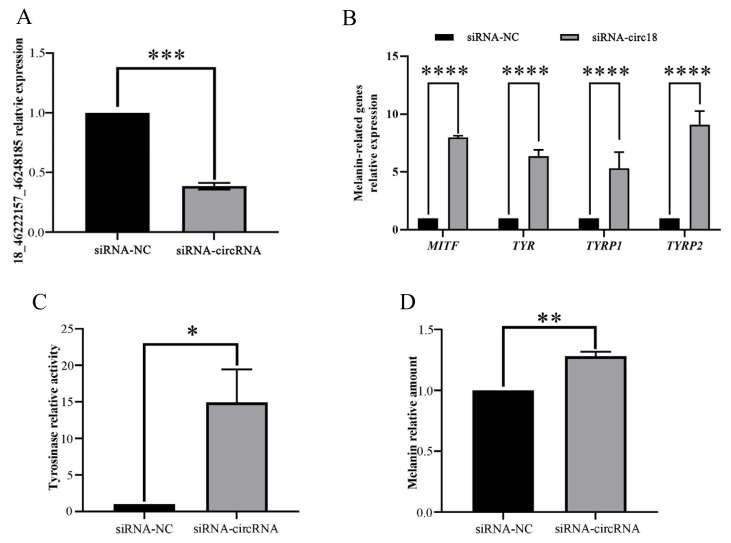
Functions of circRNA18_46222157_46248185 (named circRNA18) in goat melanocytes. The effect of siRNA-circRNA18 in melanocytes on circRNA18 expression (A), melanin-relative genes expression (B), tyrosinase activity (C), and melanin production (D). Data are shown as the means±standard deviation of the relative fold-change (Bars represent SD, n = 3 per group). * p<0.05, ** p<0.01, *** p<0.001, **** p<0.0001.

To investigate the function of circRNA18 in goat melanocytes, we constructed an interference vector (siRNA-circRNA18). qRT-PCR results showed that the expression level of circRNA18 was significantly reduced (p<0.001) upon transfection with siRNA-circRNA18 compared to that of NC group (Figure 3A). Downexpression of circRNA18 in melanocytes significantly promoted (p<0.0001) *MITF*, *TYR*, *TYRP1*, and *TYRP2* expression (Figure 3B). Furthermore, we found that the downregulation of circRNA18 in melanocytes significantly increased (p<0.05) tyrosinase activity (Figure 3C) and significantly promoted (p<0.01) melanin production (Figure 3D).

### Identification of circRNA18_46222157_46248185-miRNAs network

**Figure 4:**
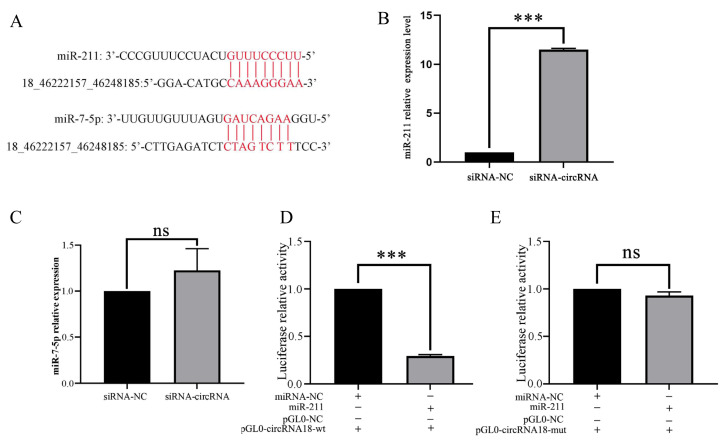
CircRNA18_46222157_46248185 inhibits miR-211 expression. (A) Bioinformatics analysis of circRNA18 targeting miR-211 and miR-7-5p. The effects of siRNA-circRNA18 on miR-211 expression (B) and miR-7-5p expression (C) were assessed. (D, E) Luciferase assay of 293T cells co-transfected with miR-211 or NC miRNA and reporter constructs containing circRNA18 wild-type (wt) or mutant (mut). Firefly luciferase activity was normalized to that of Renilla luciferase. Data are shown as the means±standard deviation of the relative fold-change (Bars represent SD, n = 3 per group). *** p<0.001, ^ns^ p>0.05.

Bioinformatics analysis unveiled the presence of putative binding sites for circRNA18 and miRNAs (miR-211 and miR-7-5p) (Figure 4A). qRT-PCR results showed that downregulation of circRNA18 significantly promoted (p<0.001) miR-211 expression (Figure 4B), however, there was no change in miR-7-5p expression (Figure 4C). Therefore, miR-211 is a potential tracking candidate. The luciferase assay results revealed that luciferase activity was significantly decreased (p<0.01) in 293T cells transfected with miR-211 and circRNA18-wt compared with that in 293T cells transfected with miR-211 and pmirGL0 dual-luciferase blank plasmid (Figure 4D). However, it was unchanged in 293T cells transfected with miR-211 and circRNA18-mut compared with the 293T cells transfected with miR-211 and pmirGL0 Dual-Luciferase blank plasmid (Figure 4E).

### Mimics-circRNA18_46222157_46248185 inhibits melanogenesis by targeting miR-211

**Figure 5:**
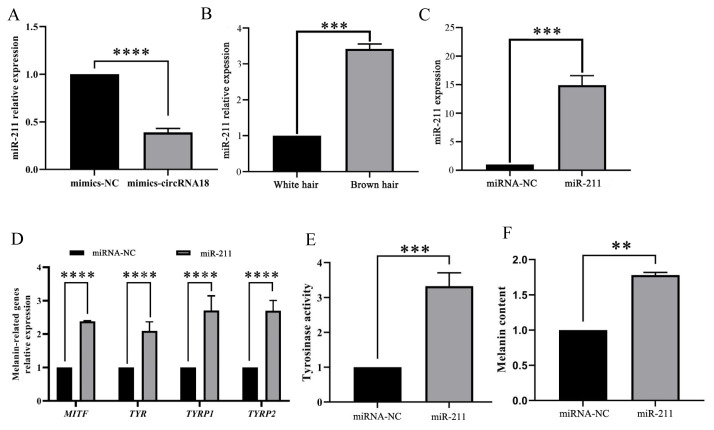
mimics-circRNA18_46222157_46248185 inhibits melanogenesis by targeting miR-211. (A) The effects of mimics-circRNA18 in melanocytes on miR-211 expression. (B) The expression trend of miR-211 in goat skins with white and brown hair. (C) The overexpression of miR-211 in melanocytes was detected using quantitative real-time polymerase chain reaction. The effects of miR-211 in melanocytes on melanin-relative genes expression (D), tyrosinase activity (E), and melanin production (F). Data are shown as the means±standard deviation of the relative fold-change (Bars represent SD, n = 3 per group). ** p<0.01, *** p<0.001, **** p<0.0001.

Mimics-circRNA18_46222157_46248185 (named mimic-circRNA18) plasmids and NC plasmids were transfected into melanocytes to determine the molecular mechanism by which circRNA18 inhibits melanogenesis. The results revealed that the overexpression of mimic-circRNA18 plasmid in melanocytes markedly decreased (p<0.0001) the expression of miR-211 (Figure 5A). qRT-PCR results showed that the expression level of miR-211 in goat skin with brown hair was significantly higher (p<0.01) than that with white hair (Figure 5B). Functional analysis showed that the overexpression of miR-211 in melanocytes (Figure 5C) significantly promoted (p<0.0001) *MITF*, *TYR*, *TYRP1*, and *TYRP2* expression (Figure 5D). Furthermore, the overexpression of miR-211 in melanocytes significantly promoted (p<0.001) tyrosinase activity (Figure 5E), and markedly increased (p< 0.01) melanin production (Figure 5F).

### miR-211 targets *EP300*

**Figure 6:**
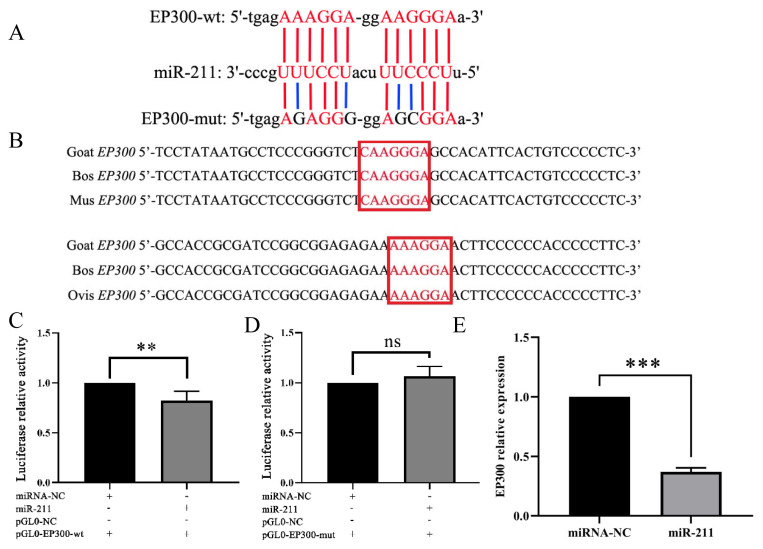
miR-211 inhibits *EP300* expression by targeting the 3′ UTR region. (A) Binding sites for miR-211 on *EP300* were predicted based on complementary base pairing. (B) Aligned *EP300* sequences around the miR-211 binding site. (C, D) Luciferase assay of 293T cells co-transfected with miR-211 or NC miRNA and reporter constructs containing *EP300* wild-type (wt) or mutant (mut). Firefly luciferase activity was normalized to that of Renilla luciferase. (E) The effects of miR-211 in melanocytes on *EP300* expression. Data represent mean±standard deviation of the relative fold-change (n = 3 per group). ** p<0.01, *** p<0.001, ^ns^ p>0.05.

Bioinformatic analysis revealed that the 3′ UTR of *EP300* has two putative miR-211 binding sites (Figure 6A) and that such putative binding sites are conserved among goat, mice, ovis and bos (Figure 6B). The luciferase assay results revealed that miR-211 expression significantly reduced (p<0.001) the luciferase activity associated with *EP300*-wt compared to that of the NC group (Figure 6C). However, it was unchanged in 293T cells transfected with miR-211 and *EP300*-mut compared with the 293T cells transfected with miR-211 and pmirGL0 Dual-Luciferase blank plasmid (Figure 6D). The qRT-PCR results showed that the overexpression of miR-211 in goat melanocytes significantly inhibited (p<0.001) *EP300* expression (Figure 6E).

### Down-regulating EP300 promotes melanin production

**Figure 7:**
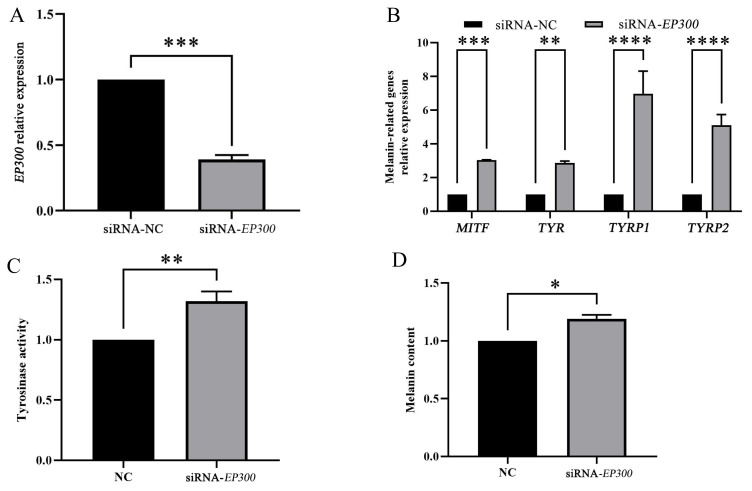
Down-regulating EP300 in goat melanocytes promotes melanin production. The effect of siRNA-*EP300* in melanocytes on *EP300* expression (A), melanin-relative genes expression (B), tyrosinase activity (C), and melanin production (D). Data represent mean±standard deviation of the relative fold-change (n = 3 per group). * p<0.05, ** p<0.01, *** p<0.001, **** p<0.0001.

We constructed interference vectors to investigate the functions of EP300 in melanocytes, and the results showed that the expression level of EP300 were significantly reduced (p<0.0001) upon transfection with siRNA-EP300 (Figure 7A). The downregulation of *EP300* in melanocytes significantly increased the transcript levels of *MITF* (p<0.001), *TYR* (p<0.01), *TYRP1* (p<0.0001), and *TYRP2* (p<0.0001) (Figure 7B). Moreover, the downregulation of *EP300* in melanocytes significantly promoted (p<0.01) tyrosinase activity (Figure 7C), and significantly increased (p<0.05) melanin production (Figure 7D).

### circRNA18_46222157_46248185 inhibits melanogenesis by targeting miR-211/*EP300* pathway

**Figure 8:**
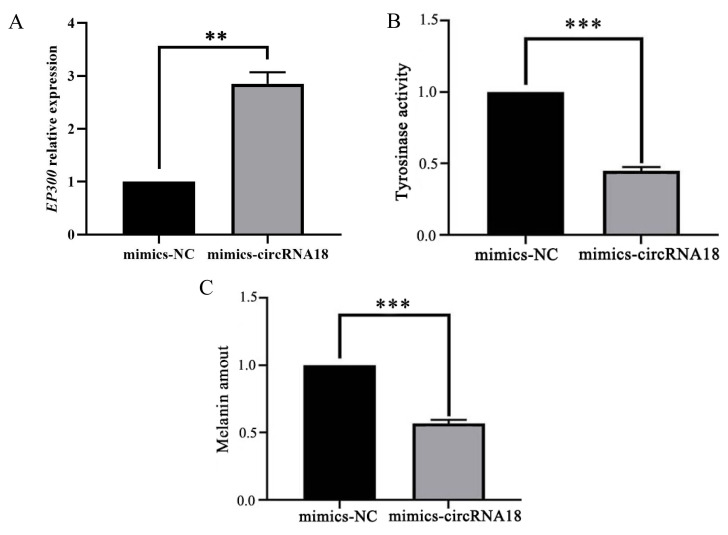
mimics-circRNA18_46222157_46248185 inhibits melanogenesis by promoting *EP300* expression. The overexpression of mimics-circRNA18 in melanocytes significantly suppressed *EP300* expression (A), markedly reduced tyrosinase activity (B), and significantly decreased melanin content (C). Data are shown as the means±standard deviation of the relative fold-change (Bars represent SD, n = 3 per group). ** p<0.01, *** p<0.001.

qRT-PCR was employed to ascertain the molecular mechanisms underlying the inhibition of melanogenesis by circRNA18 via reduction of miR-211 expression. The results showed that the overexpression of the mimics-circRNA18 in goat melanocytes significantly promoted (p<0.001) *EP300* expression (Figure 8A), significantly inhibited (p<0.001) tyrosinase activity (Figure 8B), and significantly decreased (p<0.001) melanin production (Figure 8C).

## DISCUSSION

**Figure 9:**
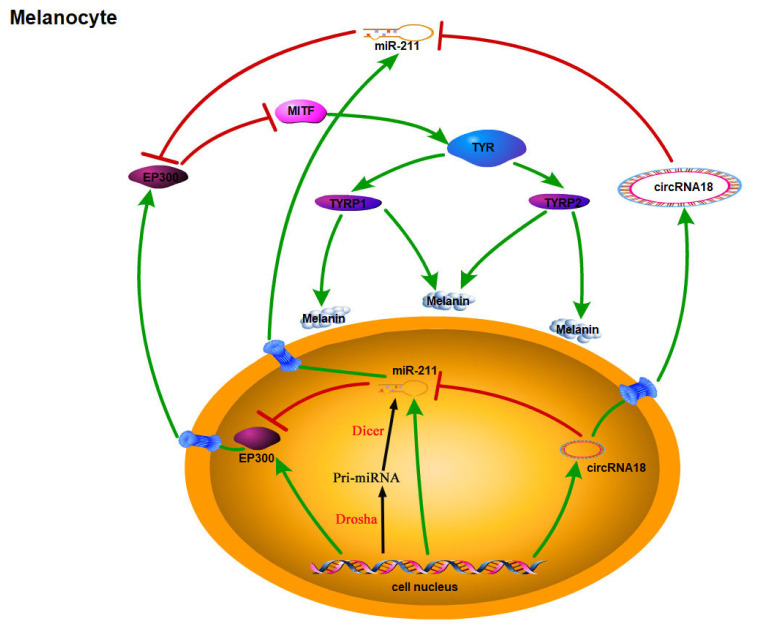
Molecular mechanism of circRNA18_46222157_46248185 inhibits melanogenesis in melanocytes.

Melanin plays a key role in determining skin and hair color in mammalian species [25,26]. circRNA-miRNA networks have been associated with various physiological processes, including bone diseases and cancer oncogenesis [17,27]. However, their roles in pigmentation of mammal remain poorly understood. In the present study, we aimed to determine the involvement of the circRNA18_46222157_46248185 (named circRNA18)-miRNAs network in this process. Mechanistic investigations unveiled that circRNA18 functions as a negative modulator of melanogenesis in goat melanocytes by attenuating miR-211 expression, whereas miR-211 fosters melanogenesis by impeding *EP300* expression. These findings are consistent with our hypothesis that circRNA18 is involved in pigmentation of goat skin as a ceRNA (Figure 9).

In contrast to conventional linear RNAs, circular RNAs are less susceptible to degradation by the RNA enzyme and exhibit enhanced stability relative to linear RNAs, underscoring their promise as biomarker candidates [13]. In the present study, the qRT-PCR results demonstrated that the expression pattern of circRNA18 in goat skins with white and brown hair corroborated the sequencing data, with no discernible impact of RNA enzyme on circRNA18 expression. The PCR results showed no positive signal in the DNA of melanocytes using specific primers, while a specific positive signal was discernible in cDNA. Based on the aforementioned research findings, it can be determined that circRNA18 is a circular RNA expressed in goat.

circRNAs can adsorb miRNAs by competing with host genes or the genes with similar target sites [14], and act as miRNA sponges to affect miRNA activity in the regulation of mRNA expression [24]. Functional analysis suggested that circRNA18 is derived from an intergenic region, and it has putative binding sites with multiple miRNAs, including miR-211 and miR-7-5p, which are known to be involved in pigmentation [19,20]. Based on this, we hypothesized that circRNA18 would be involved in pigmentation of goat skin as a ceRNA. Subsequently, we constructed an siRNA-circRNA18 and transfected into melanocytes *in vitro*, followed by the characterization of the resultant phenotypic alterations. Consistent with our hypothesis, the downexpression of circRNA18 in melanocytes significantly increased melanin production. *MITF*, a key regulator gene in melanogenesis, controls the transcription of melanogenic genes, including *TYR*, *TYRP1*, and *TYRP2* [28,29]. qRT-PCR results indicated that the downexpression of circRNA18 in melanocytes significantly promoted the transcription of these genes.

Mechanistic analyses revealed that circRNA18 inhibited the expression of miR-211 in melanocytes and the targeting relationship between circRNA18 and miR-211 was confirmed through luciferase assays. Studies have shown that miR-211 is involved in pigmentation by targeting binding RAB22A and TGFβR2 [10,30,31]. In this study, we demonstrated that the overexpression of miR-211 in melanocytes *in vitro* promoted melanogenesis. Although circRNA18 may have multiple targeted miRNAs, mechanistic analyses revealed that in melanocytes, mimics-circRNA18 inhibited melanin production by downregulating miR-211 expression. These results confirm that circRNA18 reduces pigmentation by targeting miR-211.

miRNAs can regulate gene expression by binding it’s 3′ UTR [22,23]. Our analysis revealed target binding sites between miR-211 and *EP300* in goat melanocytes, and these binding sites in *EP300* are highly conserved among goats, ovis, bos mouse. In this study, we observed that the overexpression of miR-211 in melanocytes significantly inhibited *EP300* expression, suggesting a targeted regulatory relationship between miR-211 and *EP300*. EP300, a member of the histone acetyltransferase KAT3 subfamily, is primarily located within the nuclear domain 10 structure [32]. It plays a crucial role in gene transcription modulation through the regulation of histone acetylation and crotonylation modifications [33,34]. However, their roles in melanocytes have not yet been reported. In the present study, we demonstrated that the downregulation of *EP300* expression significantly promoted melanogenesis. qRT-PCR results indicated that the downexpression of *EP300* in melanocytes markedly enhanced the transcription of melanin-relative genes. These findings reveal a novel regulatory mechanism of circRNA18 _46222157_46248185 acted as a negative regulator of melanogenesis in goat melanocytes. The architecture of circRNA18 is intricate, and this investigation solely delved into the role of circRNA18 targeting the miR-211 locus region. The functions of other structural elements remain ambiguous and warrant further exploration.

## CONCLUSION

circRNA18_46222157_46248185 acts as a negative regulator of melanogenesis in goat melanocytes by targeting miR-211/*EP300* pathway. This study provides new insights into the molecular mechanisms underlying the circRNA-miRNA network regulation of melanogenesis in mammals, and offers valuable information for guiding animal breeding efforts in the field of animal husbandry.

## Figures and Tables

**Table 1:** The sequence of primers used in this study

Primer name	Sequence (5′ to 3′)	Application
circRNA18_46222157_46248185-F	AAACTGCCAATACCCTGAA	qRT-PCR
circRNA18_46222157_46248185-R	AGGTTGCTGCCATGACTT	qRT-PCR
miR-211-F	ACACTCCAGCTGGGTTCCCTTTGTCATCCT	qRT-PCR
miR-211-RT	CTCAACTGGTGTCGTGGAGTCGGCAATTCAGTTGAGGGGCAAAG	RT-PCR
miR-7-5p-F	ACACTCCAGCTGGGTGGAAGACTAGTGATTTT	qRT-PCR
miR-7-5p-RT	CTCAACTGGTGTCGTGGAGTCGGCAATTCAGTTGAGAACAACAA	RT-PCR
common-R	TGGTGTCGTGGAGTCG	qRT-PCR
U6-F	CTCGCTTCGGCAGCACA	
CATCT	qRT-PCR	
U6-R	GTCGTATCCAGTGCAGGGTCCGAGGTATTCGCACTGGATACGACT	qRT-PCR
siRNA-*EP300*-sense (5′-3′)	CUCUGAGCGCUCUAAUAAA(dT)	siRNA
siRNA-*EP300-*antisense (5′-3′)	UUUAUUAGAGCGCUCAGAG(dT)	siRNA
TYR-F	GCTTTAGCAACTTCATGGGA	qRT-PCR
TYR-R	CTTGTTCTTCTCTGGGACAC	qRT-PCR
TYRP1-F	GCCTTCTTTCTCCCTTC	qRT-PCR
TYRP1-R	CAGACCACTCGCCATT	qRT-PCR
TYRP2-F	AGCAGACGGAACACTGGACT	qRT-PCR
TYRP2-R	GCATCTGTGGAAGGGTTGTT	qRT-PCR
EP300-F	GGGGCGACCTAATATGCAGT	qRT-PCR
EP300-R	GTCGGAATCTGGAGACCAAGG	qRT-PCR
MITF-F	TCCCAAGTCAAATGATCCAG	qRT-PCR
MITF-R	GAGCCTGCATTTCAAGTTCC	qRT-PCR
GAPDH-F	GGTTGTCTCCTGCGACTTCA	qRT-PCR
GAPDH-R	TGGTCCAGGGTTTCTTACTCC	qRT-PCR

qRT-PCR, quantitative real-time polymerase chain reaction.
